# Assessment and interpretation of the status of surgical margins following resective surgery for head and neck squamous cancer: a narrative review

**DOI:** 10.1186/s12957-025-03993-x

**Published:** 2025-08-26

**Authors:** K. Devaraja, Alok Thakar, Vinidh Paleri

**Affiliations:** 1 Department of Head and Neck Surgery, Kasturba Medical College, Manipal, Manipal Academy of Higher Education, Manipal, Udupi, Karnataka, 576104 India; 2 Department of Otorhinolaryngology and Head and Neck Surgery, All India Institute of Medical Sciences, New Delhi, 110029 India; 3 Consultant Head and Neck Surgeon, The Royal Marsden Hospitals, NHS Foundation Trust, London, SW3 6JJ UK; 4 Professor of Head and Neck Surgery, Institute of Cancer Research, London, UK

**Keywords:** Surgical margins, Oral cancer, Oropharynx, Head and neck, Frozen section, Close margin, Survival outcomes, Transoral robotic surgery

## Abstract

**Background:**

The two well-known quality-assessment metrics of head and neck oncosurgery are the status of surgical margin (SM) and lymph node yield (LNY). While the clinical importance of LNY has been well-established, several unresolved controversies around the SM have deterred its practical application.

**Methods:**

This article reviews some of the issues with the SM and ongoing efforts to improve its clinical application and reliability.

**Results:**

Several variations exist around SM, regarding its definition, designation, procurement, handling, and pathological processing, which could hinder its reliability. Until newer instruments that could improve the safety of surgical resection are validated robustly and are accessible widely, the surgeons need to adhere to the standardized approach of using the SM in clinical practice.

**Conclusions:**

Unless not available, the SM based on the surgical specimen should be given priority for all practical purposes over the tissue taken from the surgical bed; with the latter serving only as an intraoperative guide, to facilitate an appropriate margin revision whenever needed and feasible.

## Introduction

Surgery is integral to the therapeutic armamentarium in head and neck squamous cell carcinoma (HNSCC), and is particularly pertinent to oral squamous cell carcinoma(OSCC) [1–3]. The surgical excision of the primary tumor with an appropriate margin and a concurrent neck dissection, apart from eliminating the disease burden, will also provide crucial information about the pathological prognostic factors, such as the depth of invasion (DOI), worst pattern of invasion, lymphovascular invasion, perineural invasion, cervical lymph nodes and extranodal extension (ENE), that also dictate the adjuvant treatment [4–8]. Among these factors, the status of cervical lymph nodes, ENE, and DOI, owing to their stronger predictive ability, are relied upon heavily for the decision-making on the need and the type of adjuvant therapy [9, 10]. While the status of cervical lymph node in HNSCC has been a part of the American Joint Committee on Cancer (AJCC) staging system for a couple of decades, the status of ENE and DOI (for oral cancer) have also been incorporated into the latest (VIII) edition of the AJCC staging manual, thanks to the mounting evidence in the recent years on their profound impact on the overall survival (OS) [11]. There is another critical pathological prognostic factor in the surgically treated cases of HNSCC, in the form of the status of surgical margin (SM), which, like the ENE, has a direct implication on the decision about the need for adjuvant chemoradiotherapy [1, 12–15] However, despite its proven prognostic relevance, the SM is yet to find its way into the staging manual, owing to the several technical and practical hurdles that are undermining its reliability in clinical practice [16–19]. This article reviews some of the issues with the SM that are probably responsible for hindering its clinical application in the management of HNSCC.

To simplify the otherwise complex narratives around the SM, this review is organised peculiarly into four sections, with each section discussing a particular controversy, practical hurdle, or technical issue related to the SM, followed by the suggested remedial measures to overcome the respective challenge. A special emphasis is given to the emerging techniques in surgical practice that could offer potentially durable solutions for this clinical conundrum. Also, considering the pathological and prognostic heterogeneity between the HNSCC that arise from the different sites of the upper aerodigestive tract, this review focuses its discussion about the SM around the surgically treated OSCC, the principles of which may or may not be applicable to the other sites of the head and neck. However, whenever essential or feasible, we have drawn parallels to and from the surgical procedures/nuances related to the other HNSCC. Nevertheless, this review focuses on OSCC, as it is the commonest HNSCC to be treated by surgery, and the prognosis of this cancer, which is also predicted by the SM, has not yet improved to the acceptable levels. Understanding the intricacies around the SM in OSCC might lead to a better disease clearance and thus improve prognosis.

## Predicament 1: controversies related to the philosophy of SM

### 1.1. Dilemma around ‘what constitutes the SM’

A critical issue that undermines studies on the prognostic value of SM in HNSCC, including in OSCC, is the lack of a consensus on its description and standardized classification. Generally, a curative intent excision of a particular tumor would include removal of an adequate cuff of normal tissue all around it, and the adequacy of tumor clearance can only be ascertained by a histopathological examination of this normal tissue cuff around the tumor. However, there exists a lack of consensus on what constitutes an adequate SM, with two methods in use, interchangeably, to assess or designate the status of SM: the tissue sampled from the free edge of the excised specimen, called the surgical specimen margin (SSM), or the tissue sampled from the bed of the operated site, called the surgical bed margin (SBM) [16, 20–24]. One of the plausible reasons for the use of both these methods in practice is the notion that both SSM and SBM possess identical prognostic impact. However, contrary to this belief, there is a considerable body of evidence that suggests substantial differences between the two. From the practical standpoint, SBM offers a binary outcome: ‘positive (or involved)’ vs ‘negative (or clear),’ based on the presence or absence of an invasive tumor on histological assessment, and the SSM offers additional information, including the microscopic distance between the tumor and free edge of the excised surgical specimen [12, 25–28], allowing descriptors such as ‘close margin.’ The SBM, which is taken after the surgical excision of the primary tumor, as a small strip of tissue from the ‘surgeon-felt’ boundary of the tumor bed, may not necessarily be an accurate representation of the three-dimensional extent of the tumor [19]. Also, since the SBM usually involves excising an extra strip of ‘anticipated normal tissue’ from the surgical bed after the tumor removal, expectantly, the chances of finding an involved SSM in the final histopathology tend to be higher even if the SBM is negative [23, 29, 30]. By virtue of these differences between SBM and SSM, multiple studies have found SSM to have a significantly superior prognostic value than the SBM in HNSCC, including in OSCC [21, 22, 29, 31–33]. 

### Remedy: Reliance on SSM over SBM, for all practical purposes

Recognizing the substantial prognostic value of SM in oncology practice, the Union for International Cancer Control adopted a classification in 1987 to denote the presence or absence of residual (R) disease after the treatment [34, 35]. In this ‘R classification,’ a complete disease clearance is represented by 'R0,' the presence of microscopic residual tumor is designated as 'R1,' and the presence of macroscopic residual tumor is implied by 'R2' [34, 35]. Accordingly, a curative intent surgical excision can be categorized as R0 resection only when SMs from all around the surgical specimen are negative for invasive disease; and even if one of the resected SM is positive, such resection should be considered as R1. However, as mentioned above, different institutes tend to consider either one of SSM or SBM for designating the R status. This practice needs amendment, since these two SMs do not carry the same clinical and oncological implications in operated cases of HNSCC. Considering the overwhelming evidence favouring the prognostic superiority of SSM, the surgeons, as well as the multidisciplinary tumor boards, should classify the R status based on the SSM as a standard practice in HNSCC, including in OSCC, and accordingly, should prioritize their decisions based on the status of SSM, both for prognostication and for therapeutic planning. This approach is also supported by the latest practice guidelines [11, 36]. The clinical relevance of SBM in the surgical treatment of HNSCC should be limited to ascertain the completeness of disease clearance (particularly along the third dimension of a large lesion, or from a soft tissue compartment such as infratemporal fossa or skull base), and to undertake further excisions of surgical bed at doubtful or positive residual disease. However, in some of the oncosurgical procedures, which encompass piecemeal excision of the primary tumor (as in transoral surgeries for medium-to-large sized pharyngeal or laryngeal cancers or in transnasal endoscopic excision in sinonasal carcinoma), the SBM may be the only SM available for designating the R status and for the clinical decision making. In all other cases of surgically treated HNSCC, the SSM should be considered over the SBM for all practical purposes.

### 1.2. Confusion about the prognostic value of SM that is revised after being positive

Theoretically, the presence of an invasive tumor along an SM, irrespective of whether it is SSM or SBM, is bad for the prognosis as it tends to increase the chances of subsequent recurrences [37, 38]. However, in clinical practice, the prognostic relevance of the SSM varies from that of SBM due to the differences in the way surgeons handle these positive margins. While there is no doubt that a positive SM in a curative setting warrants a re-resection of the corresponding surgical bed, the feasibility and reliability of such re-resection is not always given, particularly in cases of positive SSM. The reported re-resection rates of positive SSM in oral cancer are as low as around 25–10%, and even if feasible, it is challenging to accurately re-resect the appropriate site of interest [37, 39]. Although the use of intraoperative frozen section histopathology (IFSH) can enable an immediate on-table revision of the doubtful or positive margin, due to certain reliability issues with IFSH (discussed in the subsequent section), it is not uncommon in clinical practice to end up in a situation of having a positive SM on the final histology with an inability to revise it. Irrespective of the situation , an unrevised positive SM, either SSM or SBM, is among the pathological prognostic factors with highest-risk and thus warrants an aggressive adjuvant therapy in the form of chemoradiation [21, 31]. 

On the other hand, the relevance of ‘the positive SM that has been revised until negative’ has been generally ignored, with some even considering it to be as safe as the SM that was negative upfront. However, the best available evidence indicates that the prognostic value of ‘a positive SM even after the re-resection until negative’ is not the same as that of an upfront negative SM [16, 40]. Several studies have reported ‘a need for revising the SBM’ as one of the strongest predictors of local recurrence (LR) in HNSCC after adjusting for other known predictors [29, 33, 41–47]. This was also reflected in a meta-analysis of eight eligible studies on OSCC, which showed that the ‘re-resection of an initially positive SM until it becomes negative’ was not the same as an upfront negative SM, as the re-resection did not improve local control rates significantly [48]. 

### Remedy: ‘Intermediate risk factor’ with reduced threshold for adjuvant treatment

By analysing the available literature in this regard, ‘a positive SM that has been revised until negative’ seems to fit appropriately as an intermediate pathological risk factor between the positive SSM at one end of the spectrum (as a high-risk factor), and the negative SSM at the other end (as a low-risk factor) [22, 32, 43, 48]. In such cases, although the decision about the adjuvant therapy should be guided by the presence or absence of other clinic-pathological risk factors, it could probably serve well to lower the threshold for adjuvant therapy in these cases, particularly in patients with aggressive disease variants and tolerable general profiles [33, 41, 45–47]. However, further studies are needed to validate this hypothesis and to understand the actual benefit of adjuvant treatment in such situations.

### 1.3. Inconsistencies in ‘how to designate the status of SSM’

The microscopic distance between the tumor front and the free edge of the specimen for designating a particular status to the SSM varies from institute to institute, largely due to the disparities in the literature on the ‘optimal cut-off value of this distance’ that can reliably predict the risk of the LR and local recurrence-free survival (LRFS) [49–55]. While some studies have found a margin closer than 5 millimeters (mm) to affect the LR rates [31, 39, 56], several others have failed to demonstrate a similar association [25, 27, 28, 37, 57–61]. A few studies have also reported lower cut-off values to be predictive of prognosis in OSCC: a study of 381 patients with oral tongue cancer identified SM > 2.2 mm as a better predictor of LRFS than 5 mm [51]; another study of 192 patients with oral and oropharyngeal cancers demonstrated SM of > 1.6 mm as sufficient to impart better disease-specific survival [53]; further, a larger study with a cohort of 432 patients of OSCC concluded 1 mm as sufficient margin [25]; but on the contrary, a study with 602 patients of bucco-alveolar complex reported a need of minimum margin of 5.5 mm to reduce the LRFS [49]. Table 1 summarizes some of the commonly prescribed cut-off values to designate a SM as involved, negative, or close across different research papers.

**Table 1:** Various cut-off values (microscopic distance from the tumor front to the free edge of the surgical specimen) used to designate the surgical margin in oral cancer

Status	Cut-off value(s)			
Negative	> 5 mm [39, 56, 57, 62, 63]		> 3 mm [64]	
Positive	< 2 mm [27]	Tumor [37]	< 1 mm [39, 56, 57, 63, 64]	
Close	2–5 mm [27]	0–5 mm [37]	1–5 mm [39, 55–57, 63]	1–3 mm [64]

### Remedy: Standardisation; development of prognostic models

Despite all these inconsistencies, there is a broad consensus among the head and neck surgeons that a margin of ≥ 5 mm is oncologically safe in OSCC [27, 28, 36, 37, 41, 63, 65, 66]. Accordingly, the current guidelines classify SM in oral cancer surgery to be positive, when the distance between the tumor and the free edge of the surgical specimen is < 1 mm, as close, when this distance is 1–5 mm, and as negative, when it is > 5 mm [16, 25, 33]. It is noteworthy that the cut-off values for defining the negative, close, and positive margins are different for the HNSCC arising from oropharynx, larynx, paranasal sinuses, and ear canal; and further discussion on the SM in these sites is not included in this review [52, 67]. Nevertheless, unless the objective of the study is to evaluate the impact of this distance of SM (from tumor) on the oncological outcomes in OSCC, and has sufficient statistical power and methodological considerations to study these outcomes, authors should exercise caution while publishing underpowered analyses on the significance of lower cut-off values in these tumors.

Alternatively, considering the variability in prognostic value of SM at different cut-off values, it is also worthwhile to explore the feasibility of multivariable prognostic models and nomograms in OSCC that view the SM as a continuum rather than as a categorical variable. The recently expanding applications of the machine learning/deep learning methods in medicine can aid the development and validation of such models; however, this exercise might demand comprehensive clinical data of a large number of patients, with a detailed pathological report and a long-term follow-up, with information on disease status.

### 1.4. Lack of consensus on the value of ‘close SM’ and other findings at SM

Apart from the confusion about the safe limit of minimum distance required at the surgical margin , there is also a lack of clarity in the literature on the prognostic impact of dysplasia or carcinoma-in-situ (CIS) found at the SM, and on the subsequent management of these changes [26]. Owing to the dearth of high-quality data in the literature on the clinical implications of these findings, there is a considerable variation between the institutes on how they interpret and handle such changes at the SM while making decisions on adjuvant treatment in their patients [26].

### Remedy: Assigning ‘intermediate-risk’ status to the SM with these changes

In a recently published systematic review and meta-analysis, which included 26 studies and 8435 surgically managed patients of OSCC, patients with a close SM had a lower incidence of 5-year LR rates than those with a positive SM, but a 5-year OS did not differ between the patients with close and positive margins [26]. Moreover, in this review, both the 5-year LR-free status and the OS were inferior in those with the close margins as compared to those with the negative margins, suggesting an 'intermediate status' of the close margin between that of the positive and negative SM. In other words, a close SM, like a revised SM, can be placed between the clear and involved SMs in terms of its ability to predict the LR. However, there is a need for the development and validation of multivariable prognostic models and nomograms to predict survival at various distances of SM, as these tools could provide a more realistic and clinically meaningful answer in this regard. Interestingly, a few of the published reports have also suggested a similar 'intermediate prognostic relevance' to the SMs that demonstrated either dysplasia or Cis microscopically [53, 66, 68]. Although the available evidence in this regard is not strong, there is a trend in the literature to suggest a designation of a close SM and SM with either dysplasia or Cis as an intermediate pathological risk factor, particularly in OSCC. Nevertheless, further studies are essential to validate the suggested relevancies of these SMs; until then, the final decision about the need and type of adjuvant treatment in patients with such SMs must be based on the other pathological risk factors, with a low threshold to lean towards additional therapy [25, 28, 57, 58, 65, 68]. Universal adherence to the standard practices along these lines could enable robust multicentre research outputs on these SMs and might eventually facilitate their incorporation into the staging manual.

**Fig. 1:**
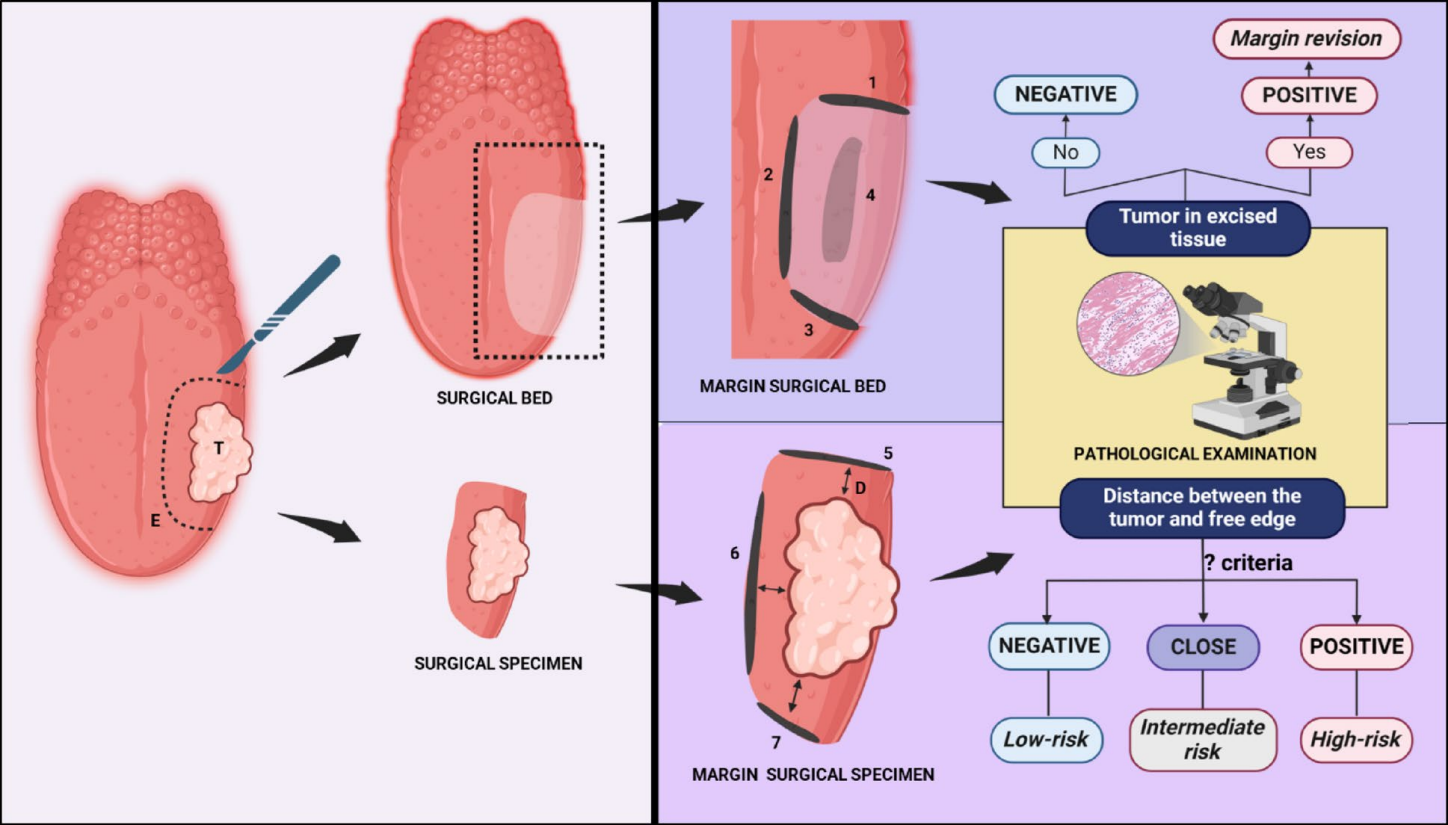
Illustration depicting the differences between two types of surgical margins and their clinical implications

The dilemma around the description and designation of the SM and their practical implications has been summarised in Fig. 1.

Key: D-Distance between the cut end of specimen and tumor; E-surgically marked excision margin; T- Tumor; 1, 2 and 3 represent surgical bed margins, respectively from the three sides of the surgical bed (along the length and breadth of the defect), and 4 is from the third dimension (depth); 5, 6 and 7 represent the surgical specimen margins corresponding respectively to 1, 2 and 3 of surgical bed margins. Note: While frozen section analysis of any of the 1 to 7 margins could be useful for intraoperative assessment and appropriate on-table revision of the microscopic residual disease, the margins 5, 6, and 7 would serve better than 1 to 4 for prognostication and decision on adjuvant therapy. [Created in BioRender. KMC, R. (2025) https://BioRender.com/l75t855].

## Predicament 2: Disease characteristics offsetting the reliability of assessment methods

Precise pre-operative assessment of the tumor extent by the clinical and radiological means and achieving an optimal intra-operative exposure (open surgery or minimally invasive approaches) of the tumor all around are the most commonly practiced surgical strategies to achieve a negative SM in HNSCC, which are subsequently expected to translate into better clinical outcomes [17, 69–71]. However, some of the disease-related factors inherent to HNSCC, particularly to OSCC, could conspire to negate these measures, in turn affecting the prognostic reliability of the SM [69, 71]. 

### 2.1. Field cancerization

OSCC is known to originate from several subclinical genetic alterations cumulated over a period of time, secondary to repetitive or persistent exposure to risk factors such as tobacco and alcohol [72–76]. In a patient with OSCC who is exposed to such risk factors, studies have shown that, the subclinical pro-carcinogenic molecular aberrations can be seen in surrounding mucosal field up to several centimeters away from the invasive carcinoma underneath a clinically and histologically apparently normal mucosa [72–77]. Thus, by this concept of ‘field cancerisation,’ a pathological negative SM of > 5 mm may not necessarily be oncologically safe, as the tissue at this distance could still carry an increased risk of subsequent carcinogenesis [72–76]. Clinical studies have reported a strong correlation between the presence of such subclinical molecular changes in histologically normal SM and an increased risk of local failure in OSCC, and have blamed ‘field cancerisation’ for the increase in the LR [78–83]. However, the current standard practice of histopathological assessment of the SM does not account for (or evaluate) the pro-carcinogenic molecular alterations [84–87]. 

### Remedy: Molecular SM (MSM)

Identification of these molecular alterations at the margin tissue can not only enhance the prognostic value of the SM in clinical practice but could also potentially permit an appropriate re-resection or revision of ‘at-risk mucosa’ whenever feasible. Studies have shown this to be an oncologically sound approach to identify the disease-free margin, with correspondingly reduced LR rates in HNSCC [78, 82, 84, 85, 88]. The evaluation of the margin tissue for the pro-carcinogenic genetic, epigenetic, or proteomic changes that are not captured by the standard histological techniques constitutes the foundation of 'MSM' [89]. It includes the application of immunostaining or DNA-based techniques, such as in situ hybridization or polymerase chain reaction, to identify the well-known molecular signatures that could improve the prognostic ability of SM [84, 86, 88]. The most common molecular signatures in histologically normal SM that have shown a strong association with an increased LR in OSCC are the loss of heterozygosity (LOH) of chromosome 9p21 (the gene which codes for p16), LOH of 17p13 (codes for TP53) and combination of LOH at 9p or overamplification of TP53 (reflected as overexpression of p53 on immunostaining) [78–80]. The presence of mutated TP53 in the SM has also shown its independent prognostic ability in operated cases of HNSCC [90]. In fact, of the several other markers and panels of molecular alterations being evaluated, alterations related to p53 protein and the TP53 genome seem to be the most appreciated for their potential utility as MSM [89]. However, in another report, the overexpression of a proto-oncogene, eukaryotic translation initiation factor 4E (eIF4E) in the SM, has demonstrated to have a better predictive value than the p53 immunopositivity [91]. A panel of methylated gene combinations identified at the SM has also shown to reliably predict the LR in HNSCC, independent of other known histological risk factors [82]. Overall, the MSM, with a known panel of markers, can complement the current clinic-pathological risk factors in operated cases of HNSCC [82, 84, 88]. Although the low coverage of the technique, additional time consumption, and financial constraints have been the major limiting factors to hinder the clinical application of MSM so far, with the recent advances in the field of biotechnology, MSM seems not too far from becoming a practical reality [85, 89]. 

### 2.2. Extension along the third dimension: difficulty in accessing the tumor depth

In an audit of 6.5 million patients with various solid cancers, the cancers arising from the oral cavity exhibited the highest rate of margin positivity across both sexes [92]. The SM in oral cancer surgery is more likely to fail along the deeper aspect (in the third dimension) than at the mucosal front, particularly in large lesions involving complex anatomical sites, such as buccal mucosa, retromolar trigone, tongue, and floor of mouth [71]. This is because, the OSCC involving these subsites can extend along the third dimension into the deeper muscles, soft tissues of the cheek/infratemporal fossa/neck, or into adjacent bony compartments, making it challenging at times to attain a clear margin all along the deeper extent [71]. The notion of having a margin of > 5 mm on the naked eye may not necessarily reflect an oncologically adequate or safe surgical margin along the third dimension, as the microscopic tumor extension in this dimension (along the nerves, muscles, and lymphatics) may go much beyond the visualized extent of the tumor. In fact, delineating the surgical margin along the deeper extent of the tumor spread, in its third dimension, is an entirely different facet than that of the mucosal margin [71]. Apart from the obvious constraints along the deeper planes such as ‘concealed microscopic spread,’ ‘difficultly in exposure of the deeper surgical site,’ and ‘potential proximity to the critical structures,’ the surgical resection in this plane, particularly while cutting through the elastic tissue, could also result in a ‘significant retraction of the cut tissue.’ These issues along the third dimension could negatively affect the orientation of the cut margins during grossing and their pathological assessment, eventually undermining the status of SM.

### Remedy: Alternate surgical approaches to accomplish optimal disease clearance in OSCC

To ensure optimal clearance of a large-volume tumor, particularly at its deeper front, currently, surgeons adopt one of the two alternative approaches. In the first approach, which aligns with the traditional onco-surgical practice, surgeons tend to remove one additional layer of the uninvolved structure around the tumor as a safe surgical margin. This approach is commonly followed in buccal cancer that nears or abuts the mandible but without involving it, in which case, stripping of periosteum from the adjacent mandible can ensure an adequate disease clearance. Similar approach is also followed in other areas, such as sinonasal tumors (where lamina papyracea, periorbita, cribriform plate, or dura can be removed to account for disease that respectively abuts and breaches the lamina papyracea, or the skull base) and small pharyngeal cancers (superior constrictor resection for tonsillar cancers and middle or inferior constrictor for hypopharyngeal cancers) [93, 94]. The second approach, which has gained popularity over the last few decades, is the concept of compartmental resection, wherein the entire anatomical compartment of the involved site or the whole organ is removed in toto with the tumor. The oncological relevance of this approach is rooted in the concept of ‘natural anatomical barriers’ that contain the spread of tumors outside the confines of a particular compartment, zone, or organ itself [95–97]. This concept forms the basis for ‘compartment tongue surgery’ or ‘compartment clearance of infratemporal fossa’ for the advanced OSCC involving oral tongue and gingiva-buccal complex, respectively [96–99]. Apart from accounting for the deeper microscopic spread of the tumor, these compartment surgeries, by ensuring a complete in toto clearance of the relevant compartments, can also reduce the risk of residual disease that could have otherwise resulted from a potential retraction of the tongue muscles or masticatory muscles during the conventional surgical excision. The philosophy of compartment resection can also be witnessed in several other onco-surgical procedures, including those for laryngeal cancers (entire spectrum of cordectomy and other forms of partial laryngectomy), sinonasal cancers (various maxillectomies and orbital exenteration), and ear cancers (temporal bone resections) [98–101]. Nevertheless, though intuitively acceptable, with favorable results in studies from a few centers, the oncological safety of some of these approaches in OSCC, as opposed to the classical approach of ‘aiming for a clear SM of ≥ 5 mm,’ continues to be a matter of debate. One of the other counteractive measures to account for the deeper microscopic spread of the tumor is the use of real-time intraoperative assessment of SM, which is discussed in the next section of this review.

## Predicament 3: Inadequacies of current intraoperative methods for assessing SM

Some of the image-based techniques such as confocal microscopy, tissue staining methods (e.g., Lugol’s iodine), narrow band imaging and other spectral imaging tools, have been explored for assessing the SM intraoperatively, as these tools could enable better demarcation of the interface between the tumor and normal tissue, allowing surgeon to achieve a safe SM away from the tumor [102]. However, most of these imaging instruments are of value only along the mucosal surfaces (two-dimensional assessment of SM), and their availability and modest diagnostic accuracy continue to be the critical barriers for their clinical application. On the other hand, IFSH, which is among the most practiced intraoperative methods for assessing the disease clearance in HNSCC, could be of more relevant to clinical practice [69, 103, 104]. Unlike the other methods described above, which facilitate identification of a safe SM away from the tumor in vivo to cut through, the IFSH, performed ex vivo on the excised tissue, is expected to help in achieving a better disease clearance by enabling an on-table (same-setting) re-resection of the SM, if found positive [19, 105–107]. The IFSH of SBM is particularly valuable in large tumors with potential microscopic extension into the third dimension (along the muscle, nerve, or other soft tissue channels) and in cases that could have satellite nodules or skip lesions away from the main tumor. In a retrospective study on oral and pharyngeal cancers, the frozen controlled re-resection of the positive SBM until negative resulted in a similar 5-year survival rate as in patients with immediate negative margins [108]. In this study, the use of IFSH led to an increase in OS rate by 2–3% among the operated oral and pharyngeal cancers. However, not many studies have been able to reproduce similar outcomes with IFSH. In fact, most of the other studies in the literature have reported poor reliability of IFSH in improving survival rates among the HNSCC, which could be attributable to a couple of practical and technical issues discussed below [29, 33, 41–47]. 

Since the IFSH involves examination of tissue taken from either SBM or SSM ex vivo, when reported positive, precise localization of the involved site on the surgical bed is prone to errors [19, 109, 110]. While some of the practices, such as the paired tagging, application of clips to the surgical field, and further re-orientation of the excised tissue fragment with sutures and colors, have all been used to counter this 'relocation errors,' they are not without practical difficulties and reliability issues [19, 24, 109–112]. Secondly, studies have reported considerable disparities between the status of ‘IFSH-designated SM’ and of the ‘final pathological classification’ (after the formalin fixation) of the same margin specimen [23, 24, 33, 44, 113, 114]. In one of the studies, the false-negative rates of IFSH in OSCC were reported to be as high as 46% [33]. Furthermore, the reliability of IFSH could also be affected by the inherent drawbacks of the SBM discussed in the earlier section; and on the other hand, the IFSH on SSM comes with the risk of mis-handling of the surgical specimen, owing to the need for a time-sensitive intraoperative reporting, which in turn, could hamper the final histopathological assessment of the SSM and its reliability [115]. Lastly, studies have also reported IFSH to be cost-ineffective as a routine practice [69, 107]. Considering these drawbacks of the IFSH approach, the guidelines that support the IFSH in HNSCC have limited its utility only to rule out the possibility of an incomplete clearance in doubtful cases, to allow an appropriate on-table re-resection [116]. In other words, the current consensus calls for attaching ‘no further clinical relevance’ to the status of SM based on IFSH, and particularly downplays the significance of SMB based IFSH in planning the adjuvant therapy.

### Remedy: Newer methods for real-time intraoperative assessment of SM

With the recent advent in the field of biotechnology, several novel technology-based instruments, with different working principles, have been introduced into surgical armamentaria for the ‘real-time intraoperative margin assessment,’ and a few of these instruments have already exhibited a promising outlook on preclinical and early clinical studies [69, 70, 102]. Table 2 provides a brief summary of some of the prominent tools that are being explored for improving the intraoperative margin assessment in HNSCC. While many of these are still useful only for two-dimensional assessment of SM, a few tools that are based on spectroscopy-based molecular imaging technology and mass spectrometry-based molecular profiling can be used for delineating the SM even along the third dimension.

**Table 2:** Prominent technology-based devices that are being explored for IMA in HNSCC

Sl no	Name	Technology	Use in HNSCC
1	Narrow band imaging	uses a high-resolution endoscope and a filter to narrow white light into distinct wavelengths of light (415 nm blue and 540 nm green) that limits penetrance to the mucosal surface for improved visualization of vessels within	[117–120]
2	Elastic scattering spectroscopy	point spectroscopic measurement of cellular and subcellular morphological features (scattering spectral signatures) with a depth of penetration of 0.5 millimeters	[121]
3	Autofluorescence	non-invasive handpiece-based illumination with bright blue light (400–460 nm), enabling direct visualization of the mucosa in real time	[122, 123]
4	Portable 3D ultrasound system	uses 3D probe, electromagnetic or optical tracker systems, or a sensorless 3D ex vivo ultrasound system	[124, 125]
5	High-resolution microendoscopy	fluorescence microscope coupled to a fiber optic imaging probe, allowing visualization of epithelial architecture and cellular morphology	[126, 127]
6	Optical coherence tomography	non-invasive modality that uses high-resolution microstructural imaging based on architectural changes with or without epithelial thickness.	[128–130]
7	Confocal reflectance microscopy	based on a signal provided by the backscattering of light from cellular and tissue structures, and image contrast is generated by differences in the refractive indexes of cellular components.	[131–133]
8	Touch Imprint Cytology	rapid histopathological method that is simpler and cheaper than frozen section	[134–137]
9	Raman spectroscopy	based on Raman Scattering - a photon in the light will interact with molecules and scatter to produce a highly detailed and specific biochemical (molecular and optical) ‘fingerprint’ of the matter	[138–142]
10	Matrix-assisted laser desorption ionization	perform tissue microsampling and real-time molecular profiling by mass spectrometry when coupled with a special laser microprobe	[143, 144]
11	intelligent Knife	aerosol generated by the diathermy is aspirated into a dedicated mass spectrometer for analysis to classify the dissected tissue as healthy or not.	[145–147]

Spectrometry can identify metabolites and variations in metabolic processes inside a tissue, and since the aberrant metabolic process is one of the characteristic features of cancer, spectrometry-based techniques can readily differentiate the tumor tissue from other tissues purely based on the profiles of its metabolites [148]. Intelligent knife (iKnife) (Waters Corp, Milford, Massachusetts, United States), which works on the principle of rapid evaporative ionization mass spectrometry (REIMS), is one of the most promising emerging techniques of real-time margin assessment [70, 144]. In this technology, the aerosol generated by the monopolar or bipolar diathermy during surgical excision is aspirated into a dedicated mass spectrometer for analysis, and the derived lipid profiles will be matched against a database of histologically annotated reference spectra by appropriate statistical analysis to classify the dissected tissue as healthy or not [70]. The iKnife has demonstrated a commanding ability to discriminate the normal tissue from a premalignant lesion and invasive cancer with high accuracy (with 100% sensitivity and specificity) in different types of cancers, including OSCC [145, 147, 149–157]. REIMS collects the mass spectral signal of the tissue under investigation at a given location by analyzing the lipids and small molecules present within surgical smoke without any need for sample preparation, and thus, delivers instant feedback in a few seconds, making it an ideal intraoperative tool for almost real-time assessment of SM [149]. Since its introduction a decade back, iKnife has been optimized over the years for better intraoperative usability, and with the further incorporation of complex machine learning strategies, it is expected to achieve higher accuracy and precision in the days to come [147, 149, 152, 158]. 

## Predicament 4: Issues around the procurement, processing, and examination of SM

Apart from the lack of consensus in terms of operational designations and the inherent disease characteristics, a few of the technical issues related to the extraction and processing of the surgical specimen could also affect the reliable assessment of the SM. 

Firstly, the three-dimensional orientation of the excised surgical specimens of the head and neck region, which is an essential step for appropriate pathological processing, could be a challenging task by itself, particularly in composite specimens that contain muscles, fascia, nerves, and bones [17, 18]. In addition to the anatomical complexity of large composite resections, the multipart resection of the tumor and an unintended tumor fragmentation during excision could all hinder an appropriate orientation of the surgical specimen. Although each element of these soft tissue components can be tagged, inked, mounted, or sutured separately at the time of dispatch from operating rooms, the final orientation of the formalin-fixed specimen might not guarantee an accurate assessment of SM. Secondly, the procedural artifacts and the post-resection changes in the surgical specimen, such as cautery burns and specimen shrinkage, could also affect the accuracy of its assessment. Similarly, changes during specimen processing, such as crush artifacts, inadvertent sectioning of an uninked specimen, and physical loss of margin tissue, are some of the additional concerns that could affect the pathological assessment of SM and its clinical reliability [17, 18]. Third, the ability to accurately define the margin status can also be impeded by a lack of proficient interdisciplinary communication between the surgical team and pathologists [17, 18, 159]. As per a report, a discrepancy between the ‘surgeon-felt SM’ and the ‘pathologically reported SM’ was found in 93% of the patients who had undergone transoral robotic surgery for oropharyngeal cancers, which impacted their therapeutic planning [94]. Lastly, the status of SM, despite the best efforts, could remain elusive in some of the clinical scenarios, owing to any of the above-mentioned procedural or processing errors. If a particular pathological report, for any reason, is not able to indicate whether the margin in a resected surgical specimen is positive, negative, or close, the status of such SM is considered indeterminate [160]. As per a review, up to 20% of the curative resections of head and neck cancer can have an indeterminate SM [160].

### Remedy: Interdisciplinary coordination

Apart from the attempts to minimise the surgical and tissue handling artifacts, another critical factor for enhancing the reliability of SM in complex tumors like HNSCC (including OSCC) is to have an efficient scheme of communication between the surgical and pathology teams. Both ‘interdisciplinary communication’ and ‘tissue handling’ can be optimised by having a frozen section facility within- or immediately adjacent to the operating theatre complex. Such a facility not only allows the surgeon to ‘hand carry’ the surgical specimen to the pathologist (minimising the orientation and tissue handling errors) but could also facilitate an effective communication of the ‘suspicious site(s)/front(s)’ of the surgical specimen to rule out a potential close or positive margin. The ‘bread-loafing of the surgical specimen’ in the presence of both surgeon and pathologist provides the maximum opportunity to have a reliable intraoperative assessment of SM, and in addition, tends to shorten the turnaround time required for pathological processing and reporting. Considering all these advantages, institutes dealing with HNSCC must embrace such close communication facilities and workflow between the surgeons and pathologists.

**Fig. 2:**
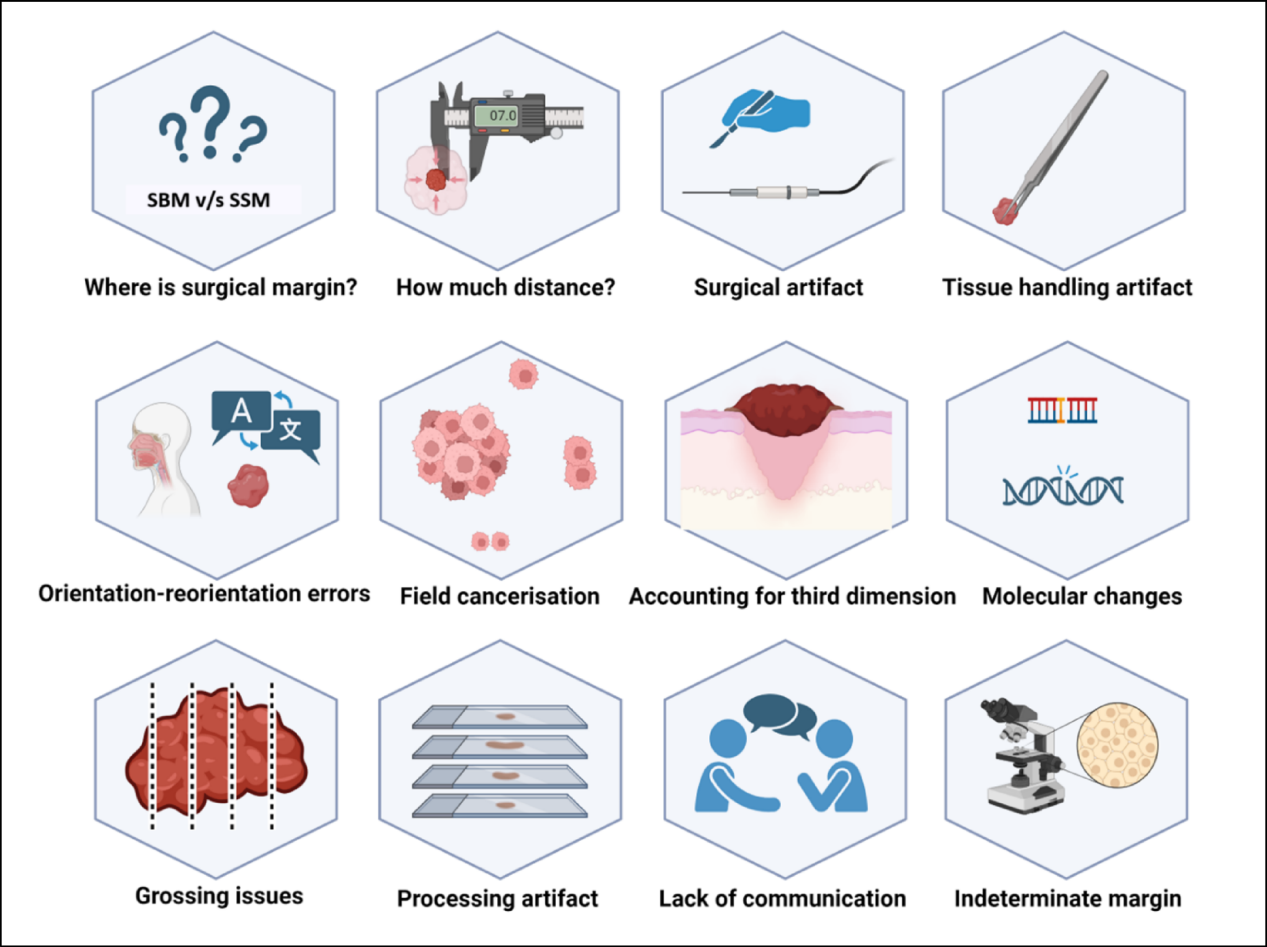
Various practical issues that could affect the reliability of the surgical margin in practice. Key: SBM- surgical bed margin and SSM- surgical specimen margin. [Created in BioRender. KMC, R. (2025) https://BioRender.com/h35f661]

To summarise, several controversies, procedural variations, and technical errors illustrated in Fig. 2 could affect the prognostic value of the status of SM, and overcoming some of these shortcomings could work to improve the practical application of SM in oncology practice.

## Conclusion

Achieving a clear SM is the prime objective of oncologic resections and is among the most important prognostic factors for disease control and cure in surgically treated OSCC. Assessment of margins at the surgical bed allows for better feasibility of accurate re-resection if required, but does not allow for discriminating between clear and close margins. Assessment of margins at the surgical specimen allows differentiation between close and clear margins and an appropriately calibrated choice of adjuvant treatment. Frozen section evaluation- despite being operator and technique-dependent and not entirely specific- remains the current standard for intraoperative margin assessment. Although newer technologies incorporating molecular imaging and metabolite-based identification, such as the iKnife, show immense potential, they are not yet a clinical reality. The standardization of the clinical practice, along with the reliance on the SSM for prognostication, whenever applicable, could enhance the reliability of SM as a pathological prognostic factor, and thus, eventually may aid in streamlining its introduction into the staging manual. However, further studies are needed to validate the relevance of re-resected SM, dysplastic SM, and MSM. It might also be worthwhile to invest in multivariable prognostic models and nomograms that account for the SM as a continuum.

## Data Availability

No datasets were generated or analysed during the current study.
